# Seminal Shedding of CMV and HIV Transmission among Men Who Have Sex with Men

**DOI:** 10.3390/ijerph120707585

**Published:** 2015-07-07

**Authors:** Sara Gianella, Konrad Scheffler, Sanjay R. Mehta, Susan J. Little, Lorri Freitas, Sheldon R. Morris, Davey M. Smith

**Affiliations:** 1 Department of Medicine, University of California, San Diego, La Jolla, CA 92037, USA; E-Mails: srmehta@ucsd.edu (S.R.M.); slittle@ucsd.edu (S.J.L.); shmorris@ucsd.edu (S.R.M.); d13smith@ucsd.edu (D.M.S.); 2 Department of Mathematical Sciences, Stellenbosch University, Stellenbosch 7602, South Africa; 3 County of San Diego Public Health Services, CA 92105, USA; E-Mail: lorri.freitas@sdcounty.ca.gov; 4 Veterans Affairs San Diego Healthcare System, San Diego, CA 92161, USA

**Keywords:** HIV transmission, cytomegalovirus, HIV epidemics, men who have sex with men

## Abstract

As in many urban areas in the United States, the largest burden of the HIV epidemic in San Diego is borne by men who have sex with men (MSM). Using data from well-characterized HIV transmitting and non-transmitting partner pairs of MSM in San Diego, we calculated the population attributable risk (PAR) of HIV transmissions for different co-infections common among MSM in this area. We found that over a third of HIV transmissions could be potentially attributed to genital shedding of cytomegalovirus (CMV) (111 transmission events), compared to 21% potentially attributed to bacterial sexually transmitted infections (STI) (62 events) and 17% to herpes simplex virus type-2 (HSV-2) (51 events). Although our study cannot infer causality between the described associations and is limited in sample size, these results suggest that interventions aimed at reducing CMV shedding might be an attractive HIV prevention strategy in populations with high prevalence of CMV co-infection.

## 1. Introduction

Most new Human Immunodeficiency Virus (HIV) infections in the United States are transmitted through male-to-male sexual contact [1]. Over 460 new HIV diagnoses were reported in San Diego County in 2013 and around three-fourths were among men who have sex with men (MSM) [2]. The true number of new infections is likely higher, since only about 85% of HIV-infected individuals are aware of their infection, despite efforts to promote universal testing and treatment [1]. Although antiretroviral therapy (ART) effectively reduces HIV transmission risk [3,4], additional strategies that target other factors associated with an increased risk of HIV transmission could improve epidemic control, including: testing and treatment of sexually transmitted infections (STI), stopping illicit substance use (e.g., methamphetamine) and enhancing condom use [1,5,6]. To use prevention resources as efficiently as possible, factors that contribute most to new HIV transmissions in the local population should be targeted preferentially.

Previous studies have found that viral, bacterial and protozoal co-infections can increase the risk of HIV transmission [1,7,8,9]. However, the proportions of HIV transmissions attributable to each of these infections differ across populations, and studies that have targeted many of these factors have proven largely unsuccessful [1,10]. One common viral co-infection that has not been evaluated systematically for contributing to HIV transmissions in a population is cytomegalovirus (CMV). In particular, shedding of CMV in semen is highly prevalent among HIV-infected MSM [11] and is associated (possibly as a causal factor) with concurrent shedding of seminal HIV RNA [7,11,12], enhanced HIV replication and up-regulation of CCR5 expression [7,13], and with transmission of HIV [14]. In particular, previous evaluations of MSM in Southern California [11,12] have found that around half (51%) of HIV-infected MSM shed CMV in their semen at any given time. Using estimates from these previously published studies in this local population, we evaluated the associations and potential contribution of CMV shedding on HIV transmissions among MSM living in San Diego County by calculating the attributable risk and comparing this effect to that of other common STI including gonorrhea, syphilis, Chlamydia and herpes simplex virus type 2 (HSV-2), which have been common targets of HIV prevention efforts [1,10,15].

## 2. Ethics Statement 

All adult subjects provided their written informed consent. No children were included in this study. The Office of Human Research Protections Program of the University of California, San Diego, approved the study (protocol #080973).

## 3. Methods and Results

We first calculated the potential contribution of seminal CMV shedding on HIV transmission in the MSM population in San Diego, California. These estimates were based on parameters measured in 46 epidemiologically and phylogenetically linked MSM pairs where one partner (the potential source partner) was HIV-infected while the other partner (the potential recipient partner) was initially HIV-uninfected [14]. These partnerships were identified through contact tracing of individuals newly diagnosed with HIV infection. Once an individual was identified as infected with HIV, they were asked about their recent sexual partners. Partnerships were defined based on timing of each individual’s HIV infection [16]. A potential source partner was defined as the individual in the named partnership who had HIV infection the longest, based on participant report and standardized serologic algorithms used in the San Diego Primary Infection Resource Consortium [17]. The potential recipient was defined as the individual who remained HIV uninfected after sexual contact with an HIV-infected partner (*i.e*., no transmission) or was HIV-infected with a HIV strain that was genetically similar to the named source partner (*i.e*., transmission). HIV transmission between named partners was confirmed by sequence analysis of infecting viral trains. In particular, phylogenetic linkage was inferred when the two viral strains were >98.5% similar in the HIV-1 *pol* coding region, as previously described [18]. HIV transmission occurred in 16 out of 30 cases (53.3%) where CMV was detectable in the potential source partners’ seminal plasma (hereafter referred to as CMV^+^) and in 4 out of 16 cases (25%) where seminal CMV was not detectable (hereafter referred to as CMV^–^). We then calculated the implications of these estimates on the transmission probabilities ( $R_{CMV}^{+}\;$ = 0.53 for CMV^+^ and $R_{CMV}^{-}\;$ = 0.25 for CMV^–^) as if they were representative of MSM partnerships in San Diego. All estimates are summarized in Table 1.

**Table 1:** Summary of associations between CMV shedding and other factors to HIV transmission among MSM in San Diego.

Parameters	Estimates	References
$I_{HIV}$: New HIV infections in San Diego country among MSM (N per year)	339	[2]
$P_{CMV}$: Frequency of seminal CMV shedding among MSM (%)	51.3%	[11,12]
$R_{CMV}^{+}$: Frequency of HIV transmission in partnerships with CMV shedding detected in the potential source partner (N (%))	16/30 (53.3%)	[14]
$R_{CMV}^{-}$: Frequency of HIV transmission in partnerships with CMV shedding not detected in the potential source partner (N (%))	4/16 (25%)	[14]
Estimated number of new HIV infections attributable to CMV (%)	125 (36.8%)	
$P_{STI}$: Frequency of bacterial STI among MSM (%)	15%	[12]
$R_{STI}^{+}$: Frequency of HIV transmission in partnerships with bacterial STI in the potential source partner (N (%))	5/5 (100%)	[14]
$R_{STI}^{-}$: Frequency of HIV transmission in partnerships without bacterial STI in the potential source partner (N (%))	15/41 (37%)	[14]
Estimated number of new HIV infections attributable to bacterial STI (%)	70 (20.6%)	
$P_{HSV}$: Frequency of HSV-2 seropositivity among MSM (%)	41%	[19]
$R_{HSV}^{+}$: Frequency of HIV transmission in partnerships with positive HSV-2 serology in the potential source partner (N (%))	8/19 (42%)	8/19 (42%)	[15]
$R_{HSV}^{-}$: Frequency of HIV transmission in partnerships with negative HSV-2 serology in the potential source partner (N (%))	7/25 (28%)	[15]
Estimated number of new HIV infections attributable to HSV-2 (%)	58 (17.1%)	

**Legend**: HIV: Human Immunodeficiency Virus, CMV: cytomegalovirus, HSV-2: herpes simplex virus type 2, N: absolute number, MSM: men who have sex with men, STI: sexually transmitted infection

We calculated the relative risk, $RR_{CMV}=\;R_{CMV}^{+}/R_{CMV}^{-}$, from the above data. Based on County of San Diego Public Health Services data, 339 new HIV infections (**I_HIV_**) were diagnosed among MSM in 2013 [2]. Based on our previously published data from MSM in San Diego, we estimated that 51% (**P_CMV_**) of HIV-infected MSM were shedding CMV in their semen at any time [11,14]. The population attributable risk (PAR) [20], expressed as a fraction of new infections, is given by: $PAR_{CMV}=\frac{P_{CMV}\left(RR_{CMV}-1\right)}{1+P_{CMV}\left(RR_{CMV}-1\right)}.$ Inserting our numbers into these equations, we estimate that 125 of the 339 (36.8%; 95% CI (calculated via the delta method): (0.66%, 72.82%)) annual new HIV infections in the San Diego MSM population may be attributable to CMV shedding.

For comparison, we repeated the calculation using the corresponding numbers for combined bacterial STI (gonorrhea, syphilis and Chlamydia). Although the importance of diagnosing and treating these bacterial infections as an intervention in the HIV epidemic is generally acknowledged [1], their combined prevalence among MSM is no more than 15% [12], which is much lower than CMV shedding (51%). Among our well-characterized MSM partner pairs, there were 5 transmissions out of 5 pairs in which the potential source partner had a bacterial STI, and 15 transmissions out of 41 pairs in which the potential source partner did not have a bacterial STI [14]. Due to the small sample size, the resulting estimate of $R_{STI}^{+}\;$ = 100% (for the transmission probability in the presence of bacterial STI) was almost certainly an over-estimate. While this overestimate biased the estimated number of transmissions attributable to bacterial STI upwards, our calculations attributed only 70 out of 339 (20.6%; 95% CI: (5.02%, 35.67%)) transmissions to bacterial STI. Note that even the upper bound of the confidence interval is lower than the point estimate for the PAR for CMV.

Finally, we estimated the potential risk of HIV transmission attributed to HSV-2 using seroprevalence data collected from two studies on HIV-infected MSM in San Diego [15,19]. Unlike CMV, the prevalence of HSV-2 DNA shedding among MSM in San Diego was very low (around 5%) [11] and presence of HSV-2 DNA shedding in seminal plasma was not associated with increased risk of HIV transmission [14], so we only considered HSV-2 seroprevalence in estimating attributable risk of HIV transmission. Repeating the above calculation with HSV-2 seroprevalence (P_HSV_) of 41% and transmission frequencies ($R_{HSV}^{+}\;$ = 42%, $R_{HSV}^{-}\;$ = 28%) taken from [15,19], we estimated that the number of infections attributable to co-infection with HSV-2 was 58 out of 339 (17.1%; 95% CI: (0.00%, 52.16%)).

## 4. Discussion

In this study, we examined the implications of the potential association between genital shedding of CMV and HIV transmission among local MSM, as observed in our previous study [14]. Interestingly, using empirical data collected from sexually active MSM living in San Diego [11,14], we estimated that up to 37% of new HIV transmissions among this group could be attributable to CMV shedding in the genital tract. Since this was an observational study, we cannot ascribe causality, and by “attributable” we are referring to the technical sense of PAR. This means that the estimated quantity is the number of new transmissions that would be prevented through eradication of CMV shedding, only if the observed association between CMV shedding and HIV transmission is causal in nature. Scaling these numbers up to sexually active MSM across the United States, which account for around 30,000 new HIV infections each year [21], we can estimate that over 10,000 new HIV infections might be potentially associated with genital shedding of CMV. As a comparison, we estimated that no more than 21% of HIV transmissions (≈6000) among MSM in the United States were associated with bacterial STI. These observations suggest that interventions aimed at reducing CMV shedding among MSM might have an impact on the incidence of HIV in that group. However, an intervention for CMV is not yet practical, given that current anti-CMV therapies are toxic and not conducive for long-term use**,** and that the results from this study are purely observational.

Previous studies on African cohorts with high prevalence of HSV-2 co-infections have attributed a large proportion of HIV transmissions to HSV-2 co-infection (≈50% in populations with >80% HSV-2 prevalence) [22]. In our study, we estimated that only 17% of HIV transmissions among MSM in San Diego were potentially attributable to co-infections with HSV-2. This is likely because the prevalence of HSV-2 was lower in our cohort of MSM compared to most African countries, and therefore its impact on HIV transmission is likely limited.

While treatment with acyclovir reduced levels of HIV RNA in blood and genital secretions, a randomized trial with 400 mg of acyclovir twice daily did not decrease HIV-1 transmission [10], suggesting that this particular prophylaxis regimen might not be enough to completely suppress replication of HSV or that other viruses not susceptible to acyclovir, like CMV, contribute considerably to HIV transmission. Alternatively, activated T cells persist at mucosal surfaces for months after cessation of active HSV replication and may represent a residual pool of target cells for HIV-infection and replication that would not be protected by acyclovir therapy [23,24]. This analysis has some important limitations. First, our estimate is based on data collected on a limited number of HIV-infected and uninfected MSM in San Diego, which might not be generalizable to the entire population of MSM living in San Diego or in the United States. Additionally, a given transmission event may be associated (or confounded) with more than one concurrent condition (e.g., both CMV shedding and bacterial STI). In that case, our method of counting would attribute the transmission to both causes, with the implication that treating either cause would be beneficial, but treating both causes may not be more beneficial than treating only one. Our calculation also liberally assumes that the correlation between genital CMV shedding and HIV viral load is causal, and thus that an intervention to reduce CMV shedding will also reduce seminal HIV replication and transmission. Alternatively, the correlation might be due to a factor which increases both CMV shedding and HIV viral load. In this case, targeting CMV may have no effect on the risk of HIV transmission. It is also important to note that all the calculated values are only point estimates with very large variance due to the small amount of available data. While we acknowledge that the available sample sizes are too small for results to be statistically significant, the existing data are suggestive of a sizeable effect, which (if real) will have a more substantial impact than more traditional risk factors. Ultimately, a study to suppress CMV shedding in at-risk individuals will need to show effectiveness of CMV control as a prevention strategy. Lastly, our data primarily relate to cases where the source partner was not treated with ART, and currently most guidelines recommend offering ART to all HIV-infected individuals in a serodiscordant relationship, regardless of CD4 count (according to current guidelines available at aidsinfo.nih.gov, accessed December 2014). Of course, we do not suggest that treating CMV should be used to prevent HIV transmission before or instead of ART initiation. Nevertheless, a considerable proportion of HIV transmissions happen during the earliest stage of HIV infections when most potential transmitting sources are still unaware of their HIV infections and have the highest levels of HIV replication in blood plasma (average of 4.7 log_10_ HIV RNA in our studies) [14,15]. Reducing the number of sexually active MSM with seminal CMV replication (independent of HIV status) might be a safe and effective intervention in epidemics that have a high HIV incidence in MSM.

## 5. Conclusions

In summary, this study found that shedding of CMV in HIV-infected MSM could potentially contribute to a large proportion of new infections. This effect may be greater than either bacterial STI or HSV-2. Understanding the attributable risk for such co-factors in local populations and risk groups could be important for designing locally appropriate prevention strategies.
